# Comparative Effectiveness in terms of Understanding of Nutri-Score and NutrInform in Spain

**DOI:** 10.1093/eurpub/ckac129.339

**Published:** 2022-10-25

**Authors:** M Fialon, N Babio, J Salas, P Galan, E Kesse-Guyot, M Touvier, B Sarda, S Hercberg, L Nabec, C Julia

**Affiliations:** 1 Nutritional Epidemiology Research Team, University of Paris, Bobigny Cedex, France; 2 Unitat de Nutrició Humana, Universitat Rovira i Virgili, Reus, Spain; 3 Institut Investigació Sanitària Pere Virgili, Reus, Spain; 4 CIBEROBN, Instituto de Salud Carlos III, Madrid, Spain; 5 Public Health Department, Assistance Publique des Hôpitaux de Paris, Paris, France; 6 RITM, Université Paris-Saclay, Paris, France

## Abstract

**Background:**

Interpretive Front-of-Pack Labels (FoPLs) are supported by WHO as a key policy tool to promote healthy diets. At present, various FoPLs formats co-exist in the European Union (EU). However, as part of the Farm to Fork strategy, the European Commission stated it would adopt a single mandatory FoPL in 2022. The aim of this study was to analyze Spanish consumers reactions to Nutri-Score and NutrInform, two FoPLs that are currently the subject of debate in EU, testing preference through subjective understanding and perception but also performance through objective understanding of the FoPLs.

**Methods:**

The experimental study was conducted in 2021 on a representative sample of 1026 Spanish adults (50% women, mean age±SD = 46±14 years), through an online randomized questionnaire where participants were exposed to Nutri-Score or NutrInform. Performance of and preference for these two FoPLs were assessed in three food categories (Breakfast Products, Breakfast Cereals and Added Fats). Performance was tested using multivariate logistic regression while preference using principal component analysis and t-tests.

**Results:**

In terms of objective understanding, Nutri-Score was significantly associated with an increase in consumers’ ability to identify healthier food products across all food categories compared to NutrInform (OR = 19.1 [14.2-25.7], p < 0.0001). On the preference dimension, Nutri-Score was perceived as significantly easier to use and was more liked than NutrInform (standardized PCA dimension resp. 0.32±1.58 vs. -0.29±1.66, p < 0.0001 and 0.080±1.18 vs. -0.072±1.17, p = 0.039) and participants found Nutri-Score more helpful to discriminate the nutritional quality of Breakfast Products and Breakfast Cereals (resp. 1.32±1.00 vs. 1.14±1.02, p < 0.01 and 1.33±1.00 vs. 1.00±1.03, p < 0.0001).

**Conclusions:**

Results of this study provide new evidence to support Nutri-Score in comparison with the NutrInform battery, on both performance and preference aspects.

**Key messages:**

• Nutri-Score better helps participants identify healthier food products than NutrInform.

• European Commission should consider results of this study in its decision on a harmonized Front-of-Pack Label.

